# Evaluation of eight commercial dog diets

**DOI:** 10.1017/jns.2014.65

**Published:** 2014-12-30

**Authors:** Caroline Daumas, Bernard-Marie Paragon, Chantal Thorin, Lucile Martin, Henri Dumon, Samuel Ninet, Patrick Nguyen

**Affiliations:** 1 Nutrition and Endocrinology Unit, ONIRIS, National College of Veterinary Medicine, Food Science, and Engineering, Nantes, France; 2 Department of Nutrition, National Veterinary School of Alfort, France

**Keywords:** Commercial dry dog diets, Digestibility, CF, crude fat, CP, crude protein, PCA, principal component analysis

## Abstract

Estimation of the quality of commercial diets is a topic of interest for the majority of dog owners. Recently, in a French consumer association magazine, an evaluation of eight dog commercial dry diets (from super-premium, basic-nutrition, private-label and economy brands) according to several nutritional criteria was published. The aims of the study were: (1) to evaluate the apparent digestibility of these diets; (2) to score these diets according to digestibility results; and (3) to compare these data with the scoring of the magazine. Six adult Beagle dogs were enrolled for the digestibility trials. Diets were scored according to energy, crude protein and crude fat (CF) apparent digestibility coefficients, digestible protein-to-energy ratios and ash content. Each of the five criteria was scored from 4 to 20 points. The ranges of crude protein, CF, crude fibre and ash content were 20·9–30·6 %, 6·8–19·7 %, 2·2–3·3 % and 4·6–9·7 % on a DM basis, respectively. The ranges of energy, crude protein and CF apparent digestibility coefficients were 72·6–87·7 %, 70·4–82·5 % and 76·1–95·4 %, respectively. The range of the protein-to-energy ratio was 10–14 digestible crude protein per MJ metabolisable energy. Little overlap in the scoring systems was found, but the private-label brand and economy brand diets presented the lowest scores in the two systems. These results showed that the evaluation of commercial diets should take into account multiple nutritional aspects. In particular, analytical and biological (digestibility) criteria should be considered as complementary in the evaluation of dry dog commercial diets.

The pet food market has grown constantly due to the increasing popularity of companion animals. Many commercial pet foods are available with different labels and from different commercial channels. Recent surveys in France and Germany have shown that the majority of pet owners feed their dogs a commercial pet food, and between one-third and one-half of them use this type of food exclusively^(^^1^^–^^2^^)^. Dry pet foods are popular because they are easy to store and represent an economical way for feeding dogs.

Pet owners have also become more and more attentive to the selection of their pets' diets because they want to provide optimal nutrition and promote the long-term health of their pets. Indeed, pet owners frequently ask how pet food is made or how to assess the quality and safety of a pet food product and which criteria are the most important in choosing a product. Veterinarians, considered as a valuable source of information by the consumer, are not always confident when recommending a pet food^(^^3^^)^. The evaluation of pet food products is thus always a topic of interest for owners and veterinarians.

Recently, in a study published in a French consumer association magazine^(^^4^^)^, eight commercial dry dog diets were scored (from super-premium brands, basic-nutrition brands, private-label brands and economy brands) according to several criteria, including nutrient content based on results of laboratory analysis.

However, the digestibility of commercial pet food is also an important criterion contributing to the variability of product quality. Although digestibility can be accurately measured using controlled digestibility trials, this criterion is sometimes indirectly estimated by pet owner from the appearance and consistency of faeces.

Therefore, the purpose of the present study was to evaluate the apparent digestibility coefficients of these eight dry dog diets in order to create a new scoring system according to the digestibility results and compare these results with the scoring of the previous study. In addition, we intended to investigate potential relationships between the variables used in the two studies.

## Experimental methods

### Animals

Six neutered adult Beagle dogs (1·5 years old each; four females and two males; weighing 8·6 (0·9) kg each and with body condition scores of 5 of 9) were enrolled in the present study. The study was conducted at the Nutrition and Endocrinology Unit of the National College of Veterinary Medicine, Food Science, and Engineering, Nantes, France (Oniris). The dogs were housed individually in closed indoor enclosures. The experimental protocol was in accordance with the European Union guidelines and was approved by the Animal Use and Care Advisory Committee of Nantes Veterinary School.

### Diets

The eight commercial dry dog diets were chosen from different commercial channels: diets G and H for the super-premium brand, diets A, B, E and F for the basic-nutrition brand, diet C for the private-label brand and diet D for the economy brand. The nutrient content of the eight commercial diets was previously analysed by an independent laboratory for the magazine study^(^^4^^)^. Then these diets were ranked on a 100 point-scale. Several criteria were scored from 0 to 20 points, and weighted as follows: protein-to-energy ratio (10 points); collagen content (8 points); crude fat (CF) content (6 points), omega 3 PUFA content (9 points); omega 6:3 ratio (6 points); vitamins and minerals content (vitamin E, 8 points; vitamin A, 4 points; ash, 6 points; potassium, 6 points; sodium, 5 points; zinc, 5 points); feeding guidelines (12 points); and mycotoxin contamination (15 points).

### Experimental design

The eight commercial diets were tested successively in a digestibility trial with a wash-out period (of at least 2 weeks) between each trial. The protocol consisted of a 7-d adaptation period followed by a 5-d collection period. In each trial, each dog was fed an amount of food previously determined to maintain its optimal body weight, and each dog had free access to water at all times.

### Measurements

In the present study, diets were scored according to energy, crude protein and CF apparent digestibility coefficients; digestible protein-to-energy ratio; and ash content. Each of the five criteria was scored from 4 to 20 points. The points were scored with the same methodology for the first four criteria: 4 points for diets presenting a value between the lowest value and the mean minus 1 sd, 8 points for diets between the mean minus 1 sd and the first quartile, 12 points for diets between the first and the third quartiles, 16 points for diets between the third quartile and the mean plus 1 sd, and 20 points for diets between the mean plus 1 sd and the highest value. For the last criterion (ash content), points were similarly scored but in the decreasing order.

### Statistical analysis

The effects of the different diets on each variable of digestibility were evaluated. Based on the design of the experiment, the statistical analysis used a linear mixed-effects model. The relationships between the different diets and the different variables of digestibility were also studied using a multifactorial analysis method (principal component analysis (PCA)). PCA, a variable reduction procedure, developed a smaller number of principal components that accounted for most of the variance in the observed variables. The active variables were the five variables of digestibility (digestibility coefficients of DM, organic matter, crude protein (CP), CF and energy and the seven variables of nutrient content (CP, CF, crude fibre, ash, omega 3 PUFA, omega 6 PUFA and hydroxyproline). The diet was integrated as a factor supplementary variable. The confidences ellipses for each diet were drawn in the scatter plot for each individual on the first-principal plane. Statistical analyses were performed using R software^(^^5^^)^ with nlme and multcomp packages.

## Results

The nutrient content obtained from an independent laboratory analysis is presented in Table 1. The metabolisable energy of the diet was calculated from the predictive equation^(^^6^^)^ and from the results of digestibility trials (Table 1).

**Table 1.** Analysed chemical composition and apparent digestibility coefficients of the eight commercial dry dog diets.

Diet	A		B		C		D		E		F		G		H	
Type of diet	BNB		BNB		PLB		EB		BNB		BNB		SPB		SPB	
Nutrient content (DM BASIS) from independent laboratory																
CP (%)	30·6		26·0		24·5		23·8		26·0		26·6		20·9		29·6	
PER (g/MJ)	18		15		15		16		16		14		12		16	
Hydroxyproline (%)	0·49		0·55		0·70		0·81		0·70		0·65		0·17		0·94	
CF (%)	16·8		13·6		12·4		6·8		13·1		12·6		16·1		19·7	
Omega 3 PUFA (%)	0·39		0·19		0·19		0·16		0·31		0·25		1·84		0·54	
Omega 6 PUFA (%)	2·82		2·12		2·02		1·64		2·93		2·61		3·57		3·37	
CFi (%)	2·2		2·7		3·3		2·6		2·4		2·5		3·3		2·4	
Ash (%)	7·3		8·4		8·7		9·7		7·3		9·2		4·6		8·4	
ME (kJ per 100 g)	1590		1550		1470		1380		1420		1510		1630		1670	
ME (kcal per 100 g)	380		370		350		330		340		360		390		400	
Digestibility results from the present study																
	M	sd	M	sd	M	sd	M	sd	M	sd	M	sd	M	sd	M	sd
dDM	82·5	2·7	78·4	1·3	69·3	2·0	66·9	1·4	84·4	2·5	79·4	1·9	82·2	1·3	80·2	1·7
dOM	87	2·2	83	1·2	75·1	1·9	74·1	1·2	87·6	1·9	83·4	1·5	84·2	1·2	84·9	1·5
dCP	82·5	3·3	79·1	1·7	70·4	4·1	72·3	1·7	81·2	3·3	78·3	2·2	80·8	2·4	79·6	2·6
dCF	93·6	1·5	90·4	1·4	85·2	2·3	76·1	4·0	95·4	0·7	90·5	0·9	92·1	0·6	94·9	0·9
dE	86·9	2·4	82·6	1·2	74·4	2·1	72·6	1·6	87·7	2·0	83	1·4	85	1·1	85·6	1·6
dPER (g/MJ)	14		13		12		14		12		12		10		13	
ME (kJ per 100 g)	1630		1420		1260		1170		1550		1420		1630		1630	
ME (kcal per 100 g)	390		340		300		280		370		340		390		390	
SCORES																
Magazine study (per 100 points)	47		57		42		45		62		58		75		48	
Digestibility study (per 100 points)	76		56		30		30		72		52		60		64	
Overall score (per 200 points)	123		113		72		75		134		100		135		112	

**Abbreviations:** BNB, basic-nutrition brand; PLB, private label brand; EB, economy brand; SPB, super premium brand; CP, crude protein; PER, protein-to-energy ratio; CF, crude fat; CFi, crude fibre; ME, metabolisable energy; dPCR, digestible protein-to-energy ratio; dDM, DM apparent digestibility coefficient; dMO, organic matter apparent digestibility coefficient; dCP crude protein apparent digestibility coefficient; dCF, crude fat apparent digestibility coefficient; dE, energy apparent digestibility coefficient; M, mean.

**Fig. 1.**
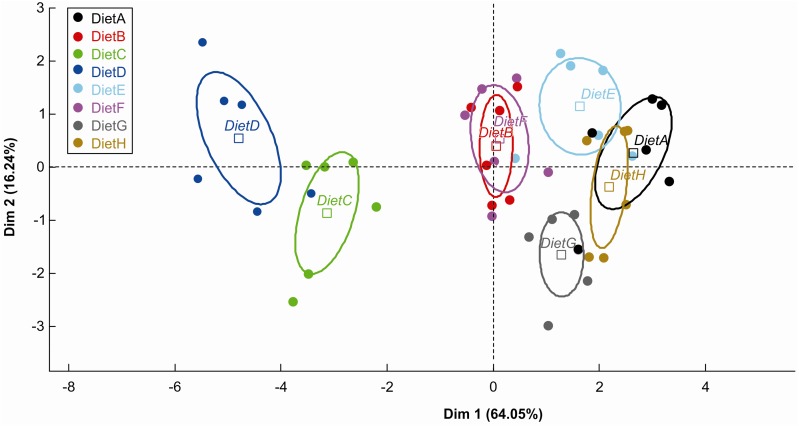
First plane for the principal component analysis (PCA) of digestibility and main nutrient content of the eight commercial dry dog diets. The diet effect on this plane is studied by the construction of 95 % confidence ellipses for each diet. • Individual dogs included in the study. □ Centre of the ellipse representing each diet.

The results obtained by linear mixed-effects models showed differences between the diets for each digestibility variable. However, PCA allowed a global visualisation of the data configuration in two dimensions (Fig. 1). The cumulative inertia on the first and second principal components reached 80·20 % of the initial variance. The main contributions to the first axis were the digestibility coefficients of DM, organic matter, CP, CF, energy and CF content, whereas the CP content contributed to the second axis. The ellipses drawn for each diet on the first plane were completely separated, except for diets A and H and diets B and F. These data indicated that most of the diets showed significant differences in properties when considering all of the digestibility components simultaneously.

The CF content and digestibility variables on the first axis exhibited a strong correlation. In contrast, on the second axis, CP content allowed discrimination between diets with similar digestibility results. For example, diets E and G showed little difference in digestibility properties and CF content but were clearly different in terms of CP content (Fig. 1).

Both ellipses that were farthest to the left of the first axis (diets C and D) corresponded with the two diets presenting the lowest scores in the magazine scoring (Fig. 1).

In the magazine's study^(^^4^^)^, scores ranked from 42 to 75 out of 100, whereas the present scores ranged from 30 to 76 out of 100. The magazine ranking was (in decreasing order): diets G, E, B, F, A, H, D and C, while the present ranking was as follows: diets A, E, H, G, B, F and D = C. Although there was no correlation between the results of the two scoring systems, diets C and D gave the lowest scores under both systems.

## Discussion

Evaluating a diet based on laboratory analysis is the first step to ensuring that the diet contains all the essential nutrients to meet the target animal's requirements. Specific laboratory analyses of the commercial product should provide levels of essential nutrients, such as PUFA, hydroxyproline and vitamins. However, based on European regulation no. 767/2009, information available on commercial dog food packaging is restricted to proximate analyses and a list of ingredients. Unfortunately, this information is not an accurate reflection of nutrient content. Estimates of CF may not include some complex lipids, and evaluation of crude fibre underestimates the level of total dietary fibre in the product. Additionally, evaluation of crude protein could be overestimated if the non-protein nitrogen portion increased. Variations in moisture content could also affect comparisons among commercial foods on an as-fed basis. Furthermore, the fact that the ingredient list is based on the weight of ingredients contributes to the confusion. An ingredient will be lower down the list, even if it contributes to a large proportion of the nutrients in the food, simply because it is dried or has low moisture content. This way of presenting nutrient analysis and the ingredient list could lead to misinterpretation when comparing two diets. Moreover, as already demonstrated, commercial dog foods can have similar guaranteed analyses and, at the same time, present great variations in apparent digestibility coefficients^(^^7^^)^.

Evaluation of apparent digestibility coefficients is thus a necessity. Commercial pet foods presenting low digestibility levels could increase fermentation by colonic bacteria and lead to excessive production of gas (flatulence) and stool and contribute to poor stool consistency. These consequences are potentially irritating for both dogs and their owners, especially for large and giant dogs, who are known for producing looser stools with higher faecal moisture than medium and small dogs^(^^8^^)^. Many factors affect the apparent digestibility of foods, including the source of ingredients^(^^9^^–^^11^^)^, fibre content^(^^12^^)^, presence of anti-nutritional factors^(^^13^^)^, ash content^(^^14^^)^ and heat treatment applied during cooking^(^^15^^)^. Consequently, digestibility could vary greatly from one brand to the next and among products within the same brand. Since there is no legal requirement to provide information on digestibility of the product, owners tend to rely on the cost of the product and the reputation of the manufacturer as criteria representing food quality. In the present study, the food presenting the highest apparent digestibility coefficients was not the most expensive (diet E); instead, it was a basic-nutrition brand. In contrast, the most expensive (diet H), a super-premium brand, did not have the highest level of crude protein or the highest digestibility coefficients. Furthermore, two diets from the same manufacturer (diets E and F) were tested and exhibited important differences in apparent digestibility. Both diets that presented the lowest digestibility results (diets C and D) differed highly from the others diets on the PCA representation, whereas the other diets were more similar. Food presenting low digestibility has to be fed in a greater amount to provide as many nutrients as a highly digestible food**.** Thus, digestibility criteria should be considered when choosing the best quality per price product.

Interestingly, the apparent digestibility coefficients of the eight commercial dog foods in the panel were well correlated with the CF content (Fig. 1). This observation was in agreement with a recent publication in blue foxes^(^^16^^)^; authors found that the apparent digestibility coefficients of the main nutrients (except for crude carbohydrates) increased along with the dietary fat level. In addition, these two species (i.e. dogs and foxes) have already been shown to have similar digestive systems, both anatomically and functionally^(^^17^^)^. In another previous study, animals were fed the same dry diets, supplemented with extra lard to deliver a total fat proportion equal to 30, 50, 70 and 80 % of the total energy of the food. The increased level of fat in the diet was shown to be related to decreases in other, less digestible nutrients, such as cellulose^(^^18^^)^ or certain sources of carbohydrates^(^^19^^)^, thereby contributing to the increased apparent digestibility coefficients of the main nutrients. In particular, the higher level of fat content in the diet increased the energy concentration of the diet and, because of the protein-sparing effects of fat, increased the apparent digestibility coefficient of protein.

In the present study, the cellulose contents were relatively comparable among diets. One of the most digestible diets (diet A) presented a high level of crude fibre, while one of the least digestible diets (diet D) a low level of crude fibre. Contrary to the hypothesis that fat has protein-sparing effects, diet H (a super-premium brand) showed low-protein apparent digestibility despite a high level of fat content in the diet. Finally, diets E and F, from the same basic-nutrition brand and the same manufacturer, showed high variations in apparent digestibility coefficients with similar CF and crude protein contents. Nevertheless, because the diets were from different brands and different manufacturers and included different sources of ingredients, the sources of variation among the eight diets were multiplied. Further studies are needed in order to evaluate the relationships between CF content and apparent digestibility coefficients in commercial dry dog diets.

The protein content contributed to the second axis of PCA (Fig. 1). This criterion discriminated among diets with the same digestibility results. This finding is not surprising considering the fact that sources of protein are the most expensive ingredient within the diet and that the digestible protein-to-energy ratio is the most variable parameter. The protein-to-energy ratio from the digestible protein level should be considered as the most relevant criterion for evaluating the protein requirements according to the type of dog food.

In the present study, the choice of the criteria used for the evaluation of the diets as well as the two methods of scoring was questionable. Notwithstanding the fact that the two systems could be improved, the comparison of the two rankings showed little overlap. The present results revealed then that these two ways of evaluation should be considered complementary.

In conclusion, although we observed certain trends in the present study, such as the increase in apparent digestibility coefficients with the increase in CF content, we have to note that there is no comprehensive list of information available to the consumer to evaluate the quality of commercial diets. A combination of laboratory analyses and estimations of digestibility coefficients is the only way to perform an accurate and complete evaluation of the quality of a commercial diet.
